# The Overlap of Small Molecule and Protein Binding Sites within Families of Protein Structures

**DOI:** 10.1371/journal.pcbi.1000668

**Published:** 2010-02-05

**Authors:** Fred P. Davis, Andrej Sali

**Affiliations:** 1 Howard Hughes Medical Institute, Janelia Farm Research Campus, Ashburn, Virginia, United States of America; 2 Department of Bioengineering and Therapeutic Sciences, Pharmaceutical Chemistry, and California Institute for Quantitative Biosciences, University of California, San Francisco, San Francisco, California, United States of America; University of California San Diego, United States of America

## Abstract

Protein–protein interactions are challenging targets for modulation by small molecules. Here, we propose an approach that harnesses the increasing structural coverage of protein complexes to identify small molecules that may target protein interactions. Specifically, we identify ligand and protein binding sites that overlap upon alignment of homologous proteins. Of the 2,619 protein structure families observed to bind proteins, 1,028 also bind small molecules (250–1000 Da), and 197 exhibit a statistically significant (p<0.01) overlap between ligand and protein binding positions. These “bi-functional positions”, which bind both ligands and proteins, are particularly enriched in tyrosine and tryptophan residues, similar to “energetic hotspots” described previously, and are significantly less conserved than mono-functional and solvent exposed positions. Homology transfer identifies ligands whose binding sites overlap at least 20% of the protein interface for 35% of domain–domain and 45% of domain–peptide mediated interactions. The analysis recovered known small-molecule modulators of protein interactions as well as predicted new interaction targets based on the sequence similarity of ligand binding sites. We illustrate the predictive utility of the method by suggesting structural mechanisms for the effects of sanglifehrin A on HIV virion production, bepridil on the cellular entry of anthrax edema factor, and fusicoccin on vertebrate developmental pathways. The results, available at http://pibase.janelia.org, represent a comprehensive collection of structurally characterized modulators of protein interactions, and suggest that homologous structures are a useful resource for the rational design of interaction modulators.

## Author Summary

Proteins function through their interactions with other biological molecules, including other proteins. Often times, these interactions underlie cellular processes that go awry in disease. Therefore, modulating these interactions with small molecules is an active area of research for new drugs to treat diseases and new chemical tools to dissect cellular interaction networks. However, targeting protein–protein interactions has proven to be more challenging than the typical drug targets found on individual proteins. Here, we present a computational approach that aims to help in this challenge by identifying regions of protein–protein interfaces that may be amenable to targeting by small molecules. Through a comprehensive analysis of all known protein structures, we identify closely related proteins that in one case bind a protein and in another case bind a small molecule. We find that a significant number of protein–protein interactions occur through surface regions that bind small molecules in related proteins. These “bi-functional” positions, which can bind both proteins and ligands, will serve as an additional piece of structural information that can aid experimentalists in developing small molecules that modulate protein interactions.

## Introduction

Protein–protein interactions are a broad class of therapeutic and chemical biology targets [1]. Traditionally these targets were thought to be refractory to small molecule modulation. However, recent efforts have led to interaction modulators that are valuable tools in mapping signalling networks and are entering clinical trials for therapeutic use [2]. Although natural substrates often serve as guides for rational drug design, such information is rarely available for protein–protein interfaces [3]. Here we attempt to provide such a starting point through a structural analysis of known protein and ligand binding sites. We posit that although ligands that are known to bind to specific protein–protein interfaces are rare, examples of ligands that bind to corresponding positions in homologous proteins may be available. These homologous sites, and the ligands they bind, may serve as starting points for rationally designing small molecule modulators of protein interactions.

The physicochemical, geometric, and evolutionary properties of ligand and protein binding sites have been extensively studied by analyzing three-dimensional protein structures [4]–[6]. On average, protein interfaces are relatively planar, more physically adaptable, and much larger than the small, rigid, pockets that bind small molecules [5],[7]. Despite the large total surface area of protein interfaces, a small subset of these residues, termed ‘hotspots’, contribute disproportionately to the affinity of protein–protein interactions [8]–[10]. Small molecules that target these hotspots have been found to effectively compete against proteins in binding events [11].

The computational methods developed for traditional rational drug design, such as pocket detection and virtual screening, have also been applied to identify small molecules modulators of protein interactions. The methods are frequently adapted to the unique properties of protein interfaces, such as their adaptivity in forming small transient cavities that can bind small molecules [12]. This property led to the use of molecular dynamics simulations to search protein interfaces for transient pockets that are subsequently targeted by virtual screening [13]. In this study, we take a conceptually related approach that harnesses the conformational (and chemical) space sampled by homologous members of a protein family. The magnitude and direction of this evolutionary sampling has been found to correlate with the conformational space sampled physically by an individual member of a protein family [14]–[16].

Here, we perform a systematic analysis of structurally characterized ligand and protein binding sites, with a central goal of comprehensively identifying, enumerating, and describing those positions in protein structure families where both ligands and proteins have been observed to bind. We first analyze the overlap of these binding sites within protein families, characterizing the composition and conservation of these ‘bi-functional’ positions, and identifying the families in which they are more or less prevalent than expected by chance. Next, we describe protein–protein and protein–peptide interactions for which small molecules were observed to bind at corresponding or homologous positions in other protein structures. Finally, we describe known interaction modulators recovered by the analysis, and illustrate its predictive utility by suggesting structural mechanisms for the observed effects of three small molecules.

## Results

### Ligand and protein binding sites

We began by assembling a comprehensive list of protein and ligand binding sites. Protein–protein (inter-molecular domain–domain, intra-molecular domain–domain, and domain–peptide) binding sites were obtained from PIBASE (v200808) [17], based on domain boundaries and classifications from SCOP (v1.73) [18] (details in Materials and Methods). Peptide binding sites were included in the analysis because the structures of protein complexes are often solved with only the peptides that mediate the interaction, rather than the full-length protein. Ligand binding sites were obtained from LIGBASE [19], and mapped onto SCOP domains using family alignments from the ASTRAL compendium [20]. Binding sites that shared more than 90% of their corresponding alignment positions were grouped together and a representative was chosen randomly, yielding a final dataset of 35,168 ligand binding sites, 2,332 peptide binding sites, 12,015 inter-molecular domain interfaces, and 4,290 intra-molecular domain interfaces, for all of which the structure is known (Table S3). This redundancy removal procedure (Materials and Methods) partially corrects the human bias in structural coverage of proteins, protein complexes, and protein-ligand complexes. Other aspects of bias can not be corrected and therefore affect our observations; For example, the analysis is limited to those proteins, ligands, and complexes that have been structurally characterized.

### Protein families with overlapping ligand and protein binding sites

We first quantified the extent and significance of overlap between all ligand and protein binding sites observed in each protein family. The binding sites were mapped onto alignments of domain families obtained from the ASTRAL compendium [20] (Fig. S1B). This mapping procedure implicitly accounts for redundant structures, as multiple structures of the same binding site do not contribute any additional positions beyond those protein-binding or ligand-binding positions identified by the first structure. Of the 2,619 families that bind proteins, 1,028 also bind small molecules, and 736 of these have at least 5 bi-functional positions (Table S1). The overlap of ligand and protein binding sites within each family was quantified using the numbers of alignment positions at which ligands (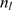), proteins (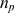), or both ligands and proteins (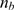) were bound, as well as the number of solvent-exposed positions (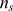).
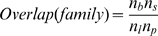
(1)


**Figure 1:**
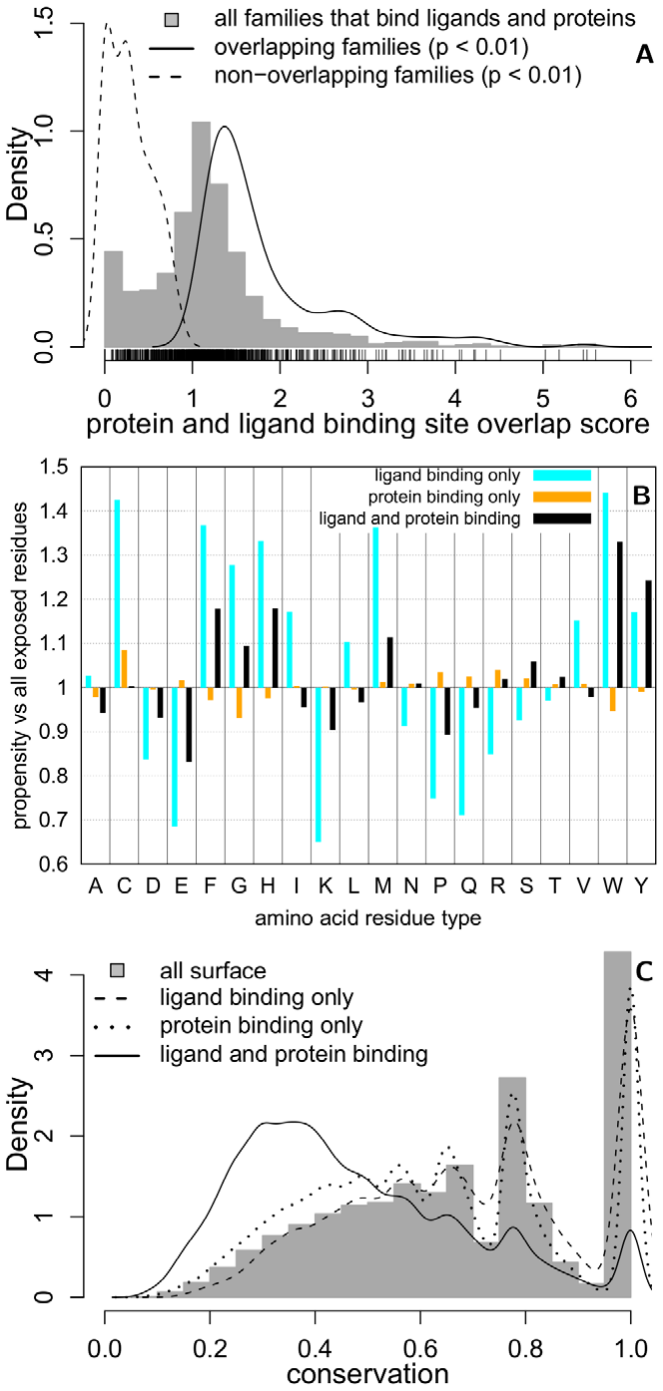
The overlap of ligand and protein binding sites within protein families. (A) The distribution of overlap scores (Eqn 1) is shown for all families that bind both ligands and proteins (grey; n = 1,028), and the subsets of families with a statistically significant overlap (p
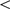
0.01; solid; n = 197) or non-overlap (p
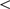
0.01; dashed; n = 113). The highest overlap score observed is 10.83 (not shown). (B) The residue type propensity (Eqn 3) and (C) conservation (Eqn 4) at alignment positions that bind both ligands and proteins (black; n = 102,436), bind ligands (cyan; n = 46,610), bind proteins (orange; n = 491,723) in comparison to all solvent-exposed residues (grey; n = 1,147,882). The statistical significance of the residue propensities was estimated by a bootstrap resampling procedure (Table S5).

An alignment position was considered solvent-exposed if at least one of the domains in the family had a residue with side-chain solvent exposure of greater than 7% at that position (MODELLER v9.4 [21]). The statistical significance (Fisher's exact one-tailed p-value) of the observed overlap for each family was assessed against a null model in which the ligand and protein binding site positions are randomly and independently placed at solvent-exposed positions (R v2.5.1, http://r-project.org). We identified 197 families with significantly more (right-tail p-val
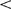
0.01), and 113 families with significantly fewer (left-tail p-val
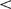
0.01), bi-functional positions than expected by chance (Fig. 1A, Table S2). These two sets of families exhibit differences in the distribution of functions as defined by SUPERFAMILY [22] (Fig. S1D). The significance of the function propensity values were estimated by a non-parametric bootstrap sampling procedure to compute 95% confidence intervals (Table S4, Materials and Methods). Families with significantly less overlap (p-val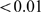) than expected by chance were enriched in Metabolism and depleted in Regulation (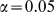). In contrast, families with significantly more overlap (p-val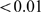) than expected by chance were depleted in Metabolism and enriched in Intracellular processes (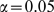). For example, ten of the overlapping families are involved in signal transduction compared to none of the non-overlapping families.

### Composition and conservation of bi-functional positions

We next asked whether the chemical or evolutionary properties of bi-functional positions were different from other positions that were part of only ligand or protein binding sites (mono-functional) or solvent-exposed. The propensities of each amino acid residue at mono-functional and bi-functional positions were calculated relative to all exposed residues, and their significance estimated by a bootstrap resampling procedure (Fig. 1B, Table S5, Materials and Methods). The magnitudes of these propensities are within the range reported in previous binding site analyses [4],[23]. The propensity of residue types that exist at the bi-functional positions are generally intermediate between those of ligand-only and protein-only positions, although they are more similar to the ligand-only positions (Fig. 1B). In particular, bi-functional positions have a higher propensity of tryptophan, histidine, and phenylalanine residues relative to both protein-binding positions and solvent exposed residues. In addition, bi-functional positions have a higher propensity for tyrosine, and slightly lower propensities for alanine, isoleucine, leucine, and valine, than either mono-functional or solvent-exposed positions.

Bi-functional positions are also significantly less conserved than mono-functional or solvent exposed positions, as measured by an entropy-based conservation score (Fig. 1C) as well as a simple count of residue types (Fig. S1E). This lower conservation was considered statistically significant (p-val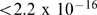) by both Kolmogorov-Smirnov and Mann-Whitney tests (Materials and Methods). Although it is difficult to precisely identify the reason for the lower conservation of bi-functional positions, one possible explanation is related to the definition of these positions. We identified bi-functional positions because they participate in different functions – ligand binding and protein binding – in different family members. These different functions might require different residue type compositions, resulting in a lower conservation score for these positions. We also observed minimal, although statistically significant (p-val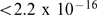), differences in conservation between mono-functional and solvent-exposed residues: ligand-only positions were more conserved than all exposed residues, which in turn were more conserved than protein-only positions. The small magnitude of the difference in conservation between mono-functional and all exposed residues is in agreement with previous findings that conservation alone is of minimal predictive use for the identification of binding sites [6].

### Protein–protein interactions with overlapping ligand binding sites

Having established that ligand and protein binding sites often overlap within protein families, we aimed to determine the utility of known ligand binding sites for targeting particular protein–protein interactions. The ligand binding sites were mapped onto individual domain–domain and domain–peptide interfaces, using ASTRAL alignments as described earlier (Fig. S1C). The overlap between each ligand binding site and protein interface was characterized by the fraction of interface residues aligned to ligand binding site residues.
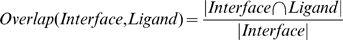
(2)


When the ligand binding site aligned to both sides of a domain–domain interface, the larger of the two overlap fractions was used as the overlap score.

The ligand binding site coverage of each protein–protein interface was summarized using two scores. First, a maximal overlap score was used to quantify the maximum overlap observed by any ligand for the protein–protein interface. Second, a cumulative overlap score was computed by simultaneously aligning all homologous ligand binding sites onto each protein–protein interface and calculating the fraction coverage. This procedure is conceptually related to fragment-based drug discovery techniques, such as tethering [24].

**Figure 2:**
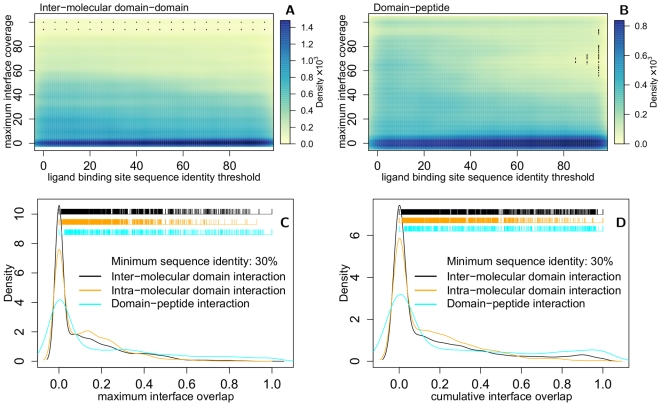
Ligand-protein binding site overlap observed at protein–protein interactions as a function of sequence identity. The maximum observed ligand binding site overlap (y-axis) for (A) inter-molecular domain–domain and (B) domain–peptide interactions, as a function of the ligand binding site sequence identity (x-axis). The densities in these plots are represented by colors that range from yellow (no density) to blue (maximum density). The (C) maximal and (D) cumulative overlap profile is shown at a minimum ligand binding site identity threshold of 30% for inter-molecular (black), intra-molecular (orange) domain–domain, and domain–peptide (cyan) interactions. Tick marks indicate interfaces that exhibit a particular level of interface coverage. The overlap score refers to the fraction of interface residues aligned to ligand binding site residues (Eqn 2).

The behavior of these overlap scores was examined as a function of the sequence identity between the ligand binding site and the corresponding positions in the interacting proteins (Fig. 2, S2). As expected, the coverage of interfaces was reduced at higher thresholds of sequence identity (Fig. 2A, 2B), and the distributions of cumulative overlap scores (Fig. S2G, S2H, S2I) exhibit a higher interface coverage than the corresponding distributions of maximum overlap scores (Fig. S2A, S2B, S2C). In addition, the domain–peptide interfaces have higher binding site overlaps (Fig. 2B), on average, than domain–domain interfaces (Fig. 2A). This observation is likely due to the smaller sizes of domain–peptide interfaces, which are thus more readily covered by small molecule binding sites.

Although the analysis suggests that most interfaces do not have a homologous ligand binding site, as seen by the main peak over an interface overlap of 0 (Fig. 2C), there are a significant number of interfaces for which overlapping homologous ligand binding sites do exist. In particular, a significant number of protein interfaces overlap with homologous ligand binding sites of greater than 30% sequence identity, previously determined to be a reliable threshold for homology transfer of ligand binding sites [25].

The systematic alignment of ligand binding sites onto protein interfaces generates a dataset useful for two primary purposes. First, it serves as a comprehensive collection of structurally characterized interaction modulators, in the cases where the ligand binding domain is identical to the sequence involved in the protein interaction (Table 1). Second, it serves as a set of predicted interaction modulators, where the ligand binding site itself is highly similar to the corresponding region in the target interaction, but the overall domain is only homologous, rather than identical (Table S6).

**Table 1:** Examples of ligand binding sites that align to protein–protein interfaces with identical or nearly identical sequences.

				Sequence identity	
Protein interaction		Ligand	Overlap	bind site	domain
*Enzyme–protein inhibitors*					
1oo9:A,B	(d) MMP-3 Catalytic Domain – N-TIMP-1	1caq:DPS	71%	100%	100%
1taw:A,B	(d) Bovine trypsin – appi	1o2h:CR3	81%	100%	100%
1a8k:A,C	(p) HIV-1 protease – ca-p2 analog	1mrw:K57	100%	100%	100%
1bzh:A,I	(p) Protein-tyrosine-phosphatase 1b – inhibitor	1g7f:INZ	100%	100%	100%
1uk4:B,H	(p) SARS proteinase 3clpro – peptide inhibitor	2alv:CY6	100%	100%	99%
1e8n:A,I	(p) Prolyl oligopeptidase – peptide	1h2y:ZPR	80%	100%	100%
1rgb:A,B	(d) Phospholipase A2 homodimer	1rgb:ELD (*)	100%	100%	100%
*Enzyme–protein substrates*					
1m9d:A,D	(d) Cyclophilin A – HIV Gag	1nmk:SFM	100%	100%	100%
1iid:A,O	(p) N-myristoyltransferase – glyaskla	2nmt:MIM	100%	100%	100%
2bgn:C,Y	(p) Dipeptidyl peptidase iv – HIV-1 tat peptide	2ajl:JNH	100%	100%	100%
1kzp:A,C	(p) Protein farnesyltransferase – k-ras4b peptide	1n94:TIN	100%	100%	98%
1q2d:A,B	(p) Histone acetyltransferase GCN5 – p53 peptide	1m1d:LYX	88%	100%	99%
1tjk:A,I	(p) Group II Phospholipase A2 – FLSTK	1fv0:9AR	86%	100%	99%
*Regulatory or structural interaction*					
1g73:A,D	(d) XIAP - BIR3	2opy:CO9	100%	100%	91%
1h1v:A,G	(d) Actin – gelsolin	1qz5:KAB	88%	100%	100%
2erj:A,D	(d) Interleukin 2–receptor	1py2:FRH	55%	100%	97%
1b6c:C,D	(d) TGF- 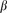 receptor – FKBP12	1bl4:AP1	76%	95%	99%
1rdt:D,E	(p) PPARG – LXXLL motif coactivator	2om9:AJA	100%	100%	99%
1mxl:C,I	(d) Cardiac troponin C–troponin I	1lxf:BEP (*)	60%	100%	100%
1g3f:A,B	(p) SMAC Diablo – XIAP BIR-3 domain	1tfq:998	100%	100%	100%
1lcj:A,B	(p) Lck SH2 domain – phosphotyrosyl peptide	1fbz:CC1	100%	95%	99%
1t4f:M,P	(p) Mdm2 – p53 peptide	1t4e:DIZ	88%	100%	100%
1f47:A,B	(p) ZipA – FtsZ fragment	1y2f:WAI	71%	100%	95%

The overlap (Eqn 2) between each ligand and protein interface is shown along with the sequence identity of the ligand binding site and the full-length domain sequence. (d) refers to inter-molecular domain–domain, (p) refers to domain–peptide interactions, and (*) indicates ligands that were present at domain interfaces.

### Recovery of known interaction modulators

To validate the accuracy of the mapping method, we checked whether known protein interaction modulators were recovered by the method. Indeed, all but one of the modulators discussed in a recent review article [2] were identified by the method: Interleukin-2 – Interleukin-2 receptor (PDB 2ERJ:A,D; 1PY2:FRH), MDM2–p53 (1T4F:M,P; 1T4E:DIZ), HPV E2–E1 helicase (1TUE:A,B; 1R6N:434), ZipA–FtsZ (1F47:A,B; 1Y2F:WAI), and TNF-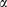 homotrimer (2TNF; 2AZ5:307). The interaction between Bcl-X–BAD (PDB 2BZW) was missed by our analysis because the ligand bound structure (2YXJ:N3C) was published too recently to be classified in the current SCOP domain database. The nearly complete recovery of known modulators suggests that the binding site data used in the analysis and the procedure used to map them operated correctly. We present additional examples of ligand binding sites that overlap interfaces to demonstrate the diversity of interactions for which ligand binding has been observed (Table 1).

### Predicted interaction modulators

Having established the accuracy of the binding site mapping, we next examined the results for their predictive utility in identifying small molecule modulators of protein interactions. Those ligand binding sites that mapped with a high sequence identity, in the context of different protein sequences, represent high confidence predictions where ligand binding may occur (Table S6). This kind of prediction is an extension of the widely used homology-transfer concept in protein function annotation [25].

### Ligand binding sites that overlap protein interfaces

**Figure 3:**
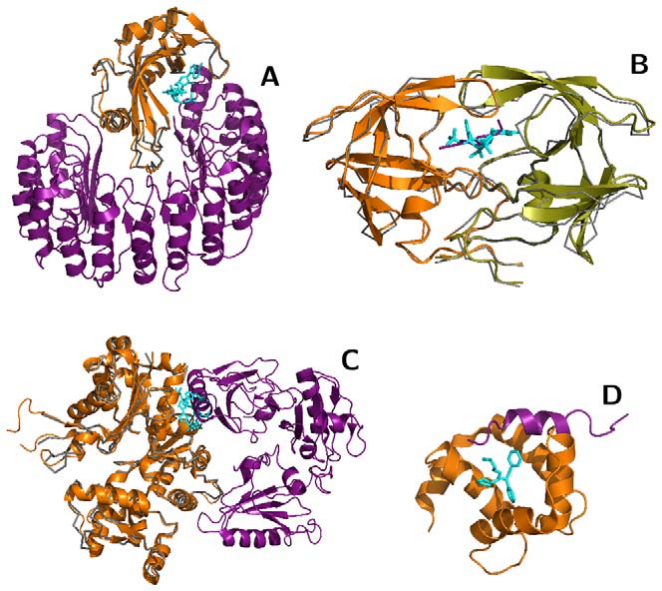
Small molecule binding sites overlapped with four broad classes of protein–protein interfaces. (A) Enzyme – protein inhibitors: *eg*, 3′-phosphothymidine (3′–5′)-pyrophosphate adenosine 3′-phosphate (PDB 1U1B:PAX) overlapped with the ribonuclease (orange, 2Q4G)–inhibitor (purple, 2Q4G) interface. (B) Enzyme–protein substrate: *eg*, Kni-577 (cyan, 1MRW:K47) bound to the HIV-protease dimer (grey backbone, 1MRW:A,B; orange, 1A8K:A,B) at the same positions as its peptide substrate (purple, 1A8K:C). (C) Structural or regulatory interfaces: *eg*, kabiramide-C (cyan, 1QZ5:KAB) bound to Actin (grey backbone, 1QZ5:A; orange, 1H1V:A) at the same position as Gelsolin (purple, 1H1V:G). (D) Several ligands complemented protein interfaces: *eg*, bepridil (cyan, 1lxf:BEP) bound at the interface between troponin C (orange, 1LXF:C) and troponin I (purple, 1LXF:I). Figure produced by PyMOL (http://pymol.org).

The ligands identified in the analysis fell into four broad categories based on the kinds of protein–protein interactions that they overlapped (Table 1, S6). The most frequently observed category were synthetic enzyme inhibitors that overlapped with the interfaces between enzymes and their protein or peptide inhibitors. These interactions include carboxypeptidase, ribonuclease, trypsins, coagulation factors, and their protein inhibitors (Fig. 3A). The high number of ligands identified in this class is not surprising, as enzyme–inhibitor complexes are among the most extensively structurally characterized and targeted by synthetic inhibitors.

A related group of ligands overlapped with the interface of an enzyme and its natural protein or peptide substrate. This class includes ligands that bound at signaling complexes such as MDM2–p53, farnesyltransfrease–h-ras, and histone acetyltransferase–p53. An example that is used therapeutically are HIV protease inhibitors bound at the protease dimer in place of its peptide substrate (Fig. 3B). We also include enzyme homodimers in this group, such as the transketolase and the ornithine decarboxylase homodimers (Table S6).

**Figure 4:**
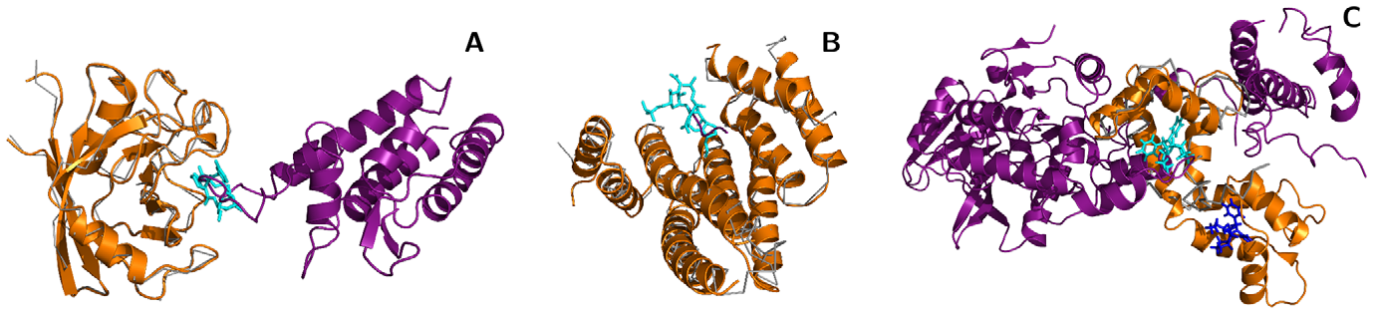
Overlapping binding sites suggest structural mechanisms for observed small molecule effects. (A) Sanglifehrin (cyan, PDB 1NMK:SFM) binds to cyclophilin A (grey, 1NMK; orange, 1AK4:A) at the same position that binds the HIV Gag capsid protein (purple, 1AK4:D). (B) Fusicoccin (cyan, 1O9E:FSC) binds to a region of the plant 14-3-3 protein (grey, 1O9E) that is homologous to the 14-3-3-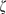 (orange, 1A38:A) binding site for phosphopeptides (purple, 1A38:P). (C) Bepridil (cyan and blue, 1LXF:BEP) binds to Troponin C (grey, 1LXF:C) at positions that are homologous to the calmodulin (orange, 1K93:D) interface for anthrax edema factor (purple, 1K93:A). Troponin C aligns to both EF-hand motifs in calmodulin: The binding site aligned with EF-motif 2 (cyan) exhibits greater overlap with the anthrax edema factor interface than EF-motif 1 (blue).

A third class of ligands overlapped with the interface of structural or regulatory protein–protein interactions. These ligands include natural toxins, such as kabiramide C bound at the actin–gelsolin interface (Fig. 3C) and fusicoccin bound at the interface of 14-3-3 proteins (Fig. 4B). This class also includes synthetic compounds such as ajulemic acid that bound at the interface of peroxisome proliferator activated recpetor gamma (PPARG) and the LXXLL coactivator (Table 1).

The fourth group of ligands were transferred from structures where they were present at domain interfaces. Although it is difficult to predict the effect of these ligands on the target interface, this group of ligands may be more likely to sterically complement protein interfaces than ligands in the other groups, which more likely sterically hinder protein interactions. This group includes elaidoylamide bound at the homodimeric interface of agkistrodotoxin Phospholipase A2 (PDB 1RGB), and bepridil bound at the interface of Troponins C and I (1LXF; Fig. 3D). Ligands in this class may be of potential use for designing chemically induced dimerization systems [26]. This technique relies on the ability of particular small molecules, such as Rapamycin and FK506, to simultaneously bind two proteins, and has been extensively used to study and control cell signaling processes. This group of ligands also slightly overlaps with the second group, as HIV protease inhibitors bind at the homodimeric protease interface (Fig. 3B). Natural ligands such as ATP, GTP, GNP also often bind at domain interfaces.

Another class of protein complexes with overlapping homologous ligand binding sites are antibody–antigen complexes. These overlaps are an expected result of the diversity of the complementary-determining regions of immunoglobulins that enable binding to virtually all proteins and small molecules.

The ligands that mapped to intra-molecular domain interfaces included natural ligands such as ATP, GTP, and Heme groups, as well as synthetic and natural toxins such as the Pulvomycin and Kirromycin antibiotics (Table S7). Since we focus on direct modulators of protein–protein interactions, we will not discuss these ligands. However, ligands that bind at intra-molecular domain interfaces may serve as logical switches in cellular signaling networks [27].

Although we observed overlaps that occur in a variety of functional classes, they can all contribute towards a structural understanding of bi-functional positions. Irrespective of the natural or synthetic source of the small molecule, or the particular functional class of protein interaction, the resulting overlaps are structurally informative for understanding what makes particular interface regions amenable to targeting by small molecules. This point can be further clarified by considering the known modulators of protein interactions that we used to test the fidelity of our mapping procedure. Although these examples involve synthetic small molecules, they have been extensively characterized structurally to understand what makes their particular binding sites amenable to targeting by small molecules [2]. Ignoring these examples because of their synthetic source would discard useful structural information.

### Predicting structural mechanisms for the observed effects of small molecules

The results also suggest possible structural mechanisms for the observed effects of small molecules. We will describe three such examples, each from a different ligand class: sanglifehrin A, bepridil, and fusicoccin. Sanglifehrin A is an immuno-suppressant, synthesized by an *Actinomycetes* species, that has been observed to reduce HIV-1 virion production [28]. Our structural analysis found that its binding site on cyclophilin A [29] overlapped completely with the complex formed by cyclophilin A and the HIV capsid [30] (Fig. 4A). This overlap suggests that sanglifehrin A competes with the HIV protein for interaction with cyclophilin A. This prediction is in agreement with biochemical evidence that describes a reduction in virion production by sanglifehrin A through a cyclophilin-dependent mechanism [28].

Fusicoccin is a toxin, synthesized by the fungus *Fusicoccum amygdali*, that disrupts protein interactions mediated by plant 14-3-3 proteins [31]. Here we observed that its ligand binding site is nearly conserved in mammalian 14-3-3 proteins and overlaps with the 14-3-3-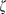
–Seretonin N-acetyltransferase and 14-3-3-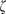
–R18 peptide interfaces (Table S6, Fig. 4B). This high level of binding site similarity suggests that fusicoccin also modulates animal 14-3-3 interactions. In fact, this modulation has been observed experimentally, with fusicoccin used as a tool to disrupt 14-3-3 interactions involved in early left-right developmental patterning in *Xenopus* [32].

Bepridil is an FDA-approved calcium channel blocker that was until recently used to treat refractory angina. Recently it was found to inhibit the cellular entry of two anthrax toxin components: the edema and lethal factors [33]. Here we observed that the troponin C binding site for bepridil [34] transfers with high sequence identity to the calmodulin–anthrax edema factor interface [35]. The ASTRAL family alignment transferred the binding site to the first calmodulin EF-hand that is not directly in contact with the edema factor. In this alignment, the binding site overlap is minimal (1 of 46 protein interface residues; Table S6, Fig. 4C) and occurs at the periphery of the interaction. However, upon visualization, it was found that the second EF-hand also aligns well with troponin C, and in this alignment the bepridil binding site directly overlaps with the edema factor interface (Fig. 4C). This alignment suggests that bepridil may disrupt the calmodulin–edema factor interaction by binding to calmodulin. This hypothesis, based on structural data alone, is in agreement with experimental findings that describe reduction in the lethality of edema factor by bepridil [33].

## Discussion

We presented a systematic analysis of protein structure families that identified bi-functional positions that bind both small molecules and proteins (Fig. 1, S1; Table S1, S2, S3). These positions were found to be less evolutionary conserved, and exhibit a different amino acid propensity, than mono-functional or other solvent exposed residues (Fig. 1, S1; Table S4, S5). Families with significantly more bi-functional positions than expected by chance were functionally enriched in intracellular processes and depleted in metabolism; families with fewer bi-functional positions were functionally enriched in metabolism and depleted in regulation (Table S4, Fig. S1D). Mapping ligands onto protein interactions by homology transfer (Fig. 2, S2) identified known (Table 1; Fig. 3) and predicted modulators of interactions (Table S6,S7), that fell into four broad categories. We illustrated the utility of the results by suggesting structural mechanisms for the observed effects of three small molecules (Fig. 4). We will now discuss future extensions to the method and its utility for modulating protein interactions by small molecules.

Our results suggest that structural data might be harnessed in a comparative fashion to characterize small molecules that target protein-protein interactions. This approach is complementary to recent computational studies that characterize known modulators of protein interactions [36] and predict small molecule mimics of interacting peptide motifs [37].

This preliminary analysis can be extended in several ways to overcome limitations inherent to the current implementation. First, the comparative basis of the method relies on the availability of homologous ligand-bound structures. Although the structural coverage of protein–ligand and protein–protein complexes continues to increase, homologous ligand binding sites are not available for the majority of protein interactions (Fig. 2, Table S3). This coverage can be improved by transferring ligand binding sites based solely on local structural similarity, rather than full-length domain similarity, as was done here. Several tools have been developed to identify local structure similarities and can be directly applied to the mapping of ligand binding sites onto protein interfaces [38]–[40].

Second, comparing the bi-functional positions to hotspot residues, that disproportionately contribute to the free energy of protein interactions, will illuminate their biophysical role [9]. Previous analysis found that hotspots are enriched in tryptophan, arginine, and tyrosine [9]. The bi-functional positions we characterize here also exhibit a strong enrichment of tyrosine and tryptophan, although arginine abundance is similar to the background of all solvent exposed positions (Fig. 1B). The bi-functional positions also exhibited significantly lower conservation than mono-functional or exposed residues (Fig. 1C). This is in contrast to hotspot residues where previous analysis has shown equivalent or slightly higher conservation than the rest of the protein surface [41]. In addition to collections of alanine scanning mutagenesis results [42], several computational techniques have been developed to predict hotspots [41],[43],[44]. Direct comparison of these datasets to bi-functional positions will help characterize their biophysical role.

Finally, although we have focused on ligands that may directly modulate, by complementing or sterically competing with, protein–protein interactions, another relevant class of interactions is allosteric regulation. Allosteric control refers to signal propagation between two distal binding sites through a network of residues that traverses a protein [45]. A portion of the ligand binding sites we found to directly overlap protein interactions may reflect allosteric sites, binding at which regulates a distal site. For example, a ligand designed to bind to the homodimeric interface of caspase-1 was found to exert allosteric control over the distal catalytic site [46]. A second potential source of allosteric interactions in our analysis is the set of 113 families that exhibited significantly less overlap than expected between ligand and protein binding sites. Further analysis should illuminate whether this observed separation between ligand and protein binding sites reflects the distal action of allosteric signaling.

### Designing small molecule modulators of protein–protein interactions

We observed that several small molecule compounds, originally designed for traditional medicinal chemistry targets such as enzyme active sites, in fact target protein interfaces. These include several FDA-approved drugs, such as bepridil that binds at the interface between Troponins C and I, and HIV protease inhibitors that bind at the dimer interface. Although these examples involve fairly small protein interfaces, this observation suggests that protein–protein interactions are not completely novel targets for medicinal chemistry, and that the chemical, biophysical, and computational experience that has been developed in traditional rational drug design may also be applicable to interaction targets.

As protein interaction networks are resolved with greater accuracy and coverage [47], small molecules become important perturbation tools to examine their functional significance. In addition, a therapeutic application that is becoming increasingly relevant is the targeting of host–pathogen protein interactions, which have been the subject of recent investigations using high-throughput experimental [48],[49] and computational [50],[51] methods. These interactions may be a valuable alternative to traditional targets for the increasingly difficult challenge of antibiotic development [52],[53]. We expect our results, available in PIBASE (http://pibase.janelia.org), to serve as a structural resource to aid in the rational design of small molecule modulators of protein–protein interactions.

## Materials and Methods

### Obtaining protein and ligand binding sites

Residues in domain–domain and domain–peptide binding sites were obtained from PIBASE v200808 [17] based on domain boundaries and classifications from SCOP v1.73 [18]. Peptides were defined as those chains at least 5 amino acid residues long that were not classified by SCOP or were classified in the “peptide or fragment” SCOP class. Binding sites were defined as residues containing at least one non-hydrogen atom within 5 Å of the interacting domain or peptide. Domain–domain interfaces were filtered using a threshold of at least 500 inter-atomic contacts at a distance threshold of 5Å (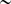
500 Å^2^ buried surface area), to remove small interfaces that are often crystallographic artifacts. A minimum domain participation of 5 residues was also imposed on domain–peptide interactions to remove small interfaces. This procedure identified 24,717 inter-molecular domain–domain, 13,228 intra-molecular domain–domain, and 6,911 domain–peptide interactions involving 2540, 1485, and 534 domain families, respectively.

Ligand binding sites were obtained from LIGBASE [19], defined as residues with at least one non-hydrogen atom within 5Å of the ligand. The analysis was restricted to PDB HETERO groups with molecular weights between 250–1000 Daltons, as this range removes crystallographic buffers and small ions present in many PDB entries, and also encompasses most orally administered drugs. MDL and CIF formatted descriptions of the ligand structures were obtained from the MSD Ligand Chemistry dictionary [54]. This procedure identified 39,085 binding sites on domains from 1,131 families.

### Removing redundant binding sites

Redundant binding sites were identified by single-linkage clustering of binding sites that shared more than 90% of their residues as measured by: (alignment positions shared by the two binding sites)/(positions in either binding site). This reduced the number of ligand binding sites from 39,085 to 35,168; peptide binding sites from 4,937 to 2,332; inter-molecular domain interfaces from 40,791 to 12,015, and intra-molecular domain interfaces from 17,863 to 4,290. The redundancy removal was performed with respect to the alignment positions, rather than amino acid sequence identity, because the binding site projection procedure relied on the alignment positions. This redundancy removal procedure aimed to reduce the effect of PDB bias in structural coverage of proteins, protein complexes, and protein-ligand complexes.

### Computing alignment position properties

The propensity of residue types in each class of position (ligand-only binding, protein-only binding, or bi-functional) was computed relative to all solvent exposed positions by counting the frequency of the 20 standard amino acid residue types:
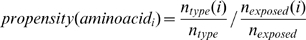
(3)


Residue types that occur more frequently at a particular binding site type than in all solvent exposed positions receive a propensity score of greater than 1, while less frequently occurring types receive a score of less than 1. The statistical significance of the propensity values was estimated by a bootstrap resampling procedure to compute 95% confidence intervals, implemented in R (http://R-project.org). Propensity values were considered significant (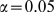) if the corresponding 95% confidence interval did not include the value of 1 [23].

The conservation of each alignment position was quantified using two scores. The first was simply the number of residue types that occurred at the position. The second was a Shannon entropy-like score that captured how non-uniform the distribution of residue type frequencies was at the position.
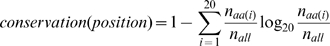
(4)


Alignment positions that contain only one kind of amino acid residue receive a conservation score of 1, while those with a uniform distribution of residue types receive a score of 0. The distributions of conservation scores for each kind of alignment position (bi-functional, ligand-only, protein-only, or all exposed residues) were compared using the Kolmogorov-Smirnov and Mann-Whitney tests, as implemented in R (http://R-project.org).

### Computing function propensities

Each family was assigned one of seven broad functions by SUPERFAMILY [22]: General, Information, Metabolism, Not Annotated, Other, Extracellular processes or Intracellular processes. The function propensities of families with significantly greater or fewer bi-functional positions than expected by chance were computed relative to the frequency of functions in all families.
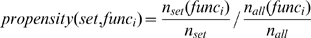
(5)


Functions that occur more frequently in a particular set of families than in all families, receive a score of greater than 1. The significance of the function propensity values was estimated by a non-parametric bootstrap resampling procedure to compute 95% confidence intervals, implemented in R (http://R-project.org). Propensity values were considered significant (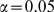) if the corresponding 95% confidence interval did not include the value of 1.

## Supporting Information

Table S1 Summary of protein and small molecule binding sites in families of protein structures. The numbers of protein families with at least 5 bi-functional positions are shown for each kind of protein interface. Bi-functional positions refer to alignment positions that bind both small molecules (250–1000 Da) and proteins.
(0.03 MB PDF)

Table S2 The ten families with the most significantly (p<0.01) higher or lower number of bi-functional positions than expected by chance. Bi-functional positions refer to alignment positions that bind both small molecules (250–1000 Da) and proteins. The significance of the overlap (Text Eqn 1) is assessed by the Fisher exact test (http://r-project.org).
(0.04 MB PDF)

Table S3 Summary of protein interactions and their overlap with aligned ligand binding sites from homologous structures. The numbers of protein interfaces with at least 20% cumulative or maximal overlap with homologous ligand binding sites are shown for each kind of protein interface. The overlap score refers to the fraction of interface residues aligned to ligand binding site residues (Text Eqn 2).
(0.03 MB PDF)

Table S4 The function propensities of families with significantly (p<0.01) higher or lower number of bi-functional positions than expected by chance. Bootstrap resampling was performed to compute 95% confidence intervals of the function propensities (Text Eqn 5). Propensities are considered significant (asterisk) at the alpha = 0.05 level if their confidence intervals do not include the value 1.
(0.03 MB PDF)

Table S5 The residue type propensity at alignment positions that bind both ligands and proteins, bind ligands, or bind proteins in comparison to all solvent-exposed residues. Bootstrap resampling was performed to compute 95% confidence intervals (CI) of the residue type propensities (Text Eqn 3). Propensities are considered significant (asterisk) at the alpha = 0.05 level if their confidence intervals do not include the value 1.
(0.03 MB PDF)

Table S6 Examples of ligand binding sites that align to protein–protein interfaces with a high sequence similarity. The overlap (Text Eqn 2) between each ligand and protein interface is shown along with the sequence identity of the ligand binding site and the full-length domain sequence. (d) refers to inter-molecular domain–domain interactions, (p) refers to domain–peptide interactions, and (*) indicates ligands that were present at domain interfaces.
(0.04 MB PDF)

Table S7 Examples of ligand binding sites that align to intra-molecular domain–domain interfaces. The overlap (Text Eqn 2) between each ligand and domain interface is shown along with the sequence identity of the ligand binding site and the full-length domain sequence.
(0.03 MB PDF)

Figure S1 Protocol for quantifying binding site overlap, functional, and evolutionary properties. (A) Ligand and protein binding sites obtained from LIGBASE and PIBASE, respectively, were mapped onto domain family alignments from the SCOP ASTRAL compendium. (B) The square labeled A is a cartoon representation of a protein domain family upon which ligand (diamonds) and protein (grey ellipses) have been mapped. These binding sites are mapped onto the ASTRAL alignment of the family and the cumulative overlap of ligand and protein binding positions is quantified. (C) The ligand binding sites are also mapped directly onto individual protein interfaces, in this case the interaction between domains A and B, and the overlap quantified. (D) The distribution of function propensities (Text Eqn 5) for significantly overlapping and non-overlapping families, as annotated by SUPERFAMILY. Function propensities were considered significant (asterisk) at the alpha = 0.05 level if the 95% confidence interval estimated by bootstrap resampling did not include the value 1 (Table S4). (E) Residue conservation of bi-functional alignment positions. The number of amino acid types observed at alignment positions that are involved in binding only ligands (dashed; n = 46,610), only proteins (double dashed; n = 491,723), or both proteins and ligands (black;n = 102,436). The distribution for all solvent exposed residues (grey; n = 1,147,882) is shown for comparison.
(0.34 MB TIF)

Figure S2 Maximum and cumulative ligand-protein binding site overlap observed at protein–protein interactions as a function of sequence identity. The maximum and cumulative observed ligand binding site overlap (y-axis) for (A,G) inter-molecular, (B,H) intra-molecular domain–domain, and (C,I) domain–peptide interactions, as a function of the ligand binding site sequence identity (x-axis). The densities in these plots are represented by colors that range from yellow (no density) to blue (maximum density). The overlap profiles are shown at minimum ligand binding site identity thresholds of (D,J) 30%, (E,K) 50%, and (F,L) 90% for inter-molecular (black), intra-molecular (orange) domain–domain, and domain–peptide (cyan) interactions. Tick marks, arranged as ‘rug plots’, represent interfaces of each type that exhibit a particular level of interface coverage. The overlap score refers to the fraction of interface residues aligned to ligand binding site residues (Text Eqn 2).
(1.87 MB TIF)
