# Supplementary material for: The Overlap of Small Molecule and Protein Binding Sites within Families of Protein Structures
Source: PLoS Comput Biol. 2010 Feb 5;6(2):e1000668. doi: 10.1371/journal.pcbi.1000668 (PMC2816688; doi:10.1371/journal.pcbi.1000668)
Supplement: Table S1 — Summary of protein and small molecule binding sites in families of protein structures. The numbers of protein families with at least 5 bi-functional positions are shown for each kind of protein interface. Bi-functional positions refer to alignment positions that bind both small molecules (250–1000 Da) and proteins. (0.03 MB PDF) [file pcbi.1000668.s001.pdf]

Table S1: **Summary of protein and small molecule binding sites in families of protein structures.**

Number of families	Total	bind small molecules	$\geq$ 5 bi-functional positions
Total	3,463	1,131	
Domain–peptide	469	232	150
Domain–domain, inter-chain	2,120	900	570
Domain–domain, intra-chain	1,189	562	356
Total protein-binding	2,619	1,028	736

The number of protein families with at least 5 bi-functional positions are shown for each kind of protein interface. Bi-functional positions refer to alignment positions that bind both small molecules (250-1000 Da) and proteins.
