# Supplementary material for: The Overlap of Small Molecule and Protein Binding Sites within Families of Protein Structures
Source: PLoS Comput Biol. 2010 Feb 5;6(2):e1000668. doi: 10.1371/journal.pcbi.1000668 (PMC2816688; doi:10.1371/journal.pcbi.1000668)
Supplement: Table S2 — The ten families with the most significantly (p<0.01) higher or lower number of bi-functional positions than expected by chance. Bi-functional positions refer to alignment positions that bind both small molecules (250–1000 Da) and proteins. The significance of the overlap (Text Eqn 1) is assessed by the Fisher exact test (http://r-project.org). (0.04 MB PDF) [file pcbi.1000668.s002.pdf]

Table S2: **The ten families with the most significantly ($p < 0.01$) higher or lower number of bi-functional positions than expected by chance.**

Family	overlap	significance
Most significant overlapping families		P-value (right)
Amylase, catalytic domain	1.35	2.2e-16
Tyrosine-dependent oxidoreductases	1.23	2.2e-16
Tandem AAA-ATPase domain	1.32	2.2e-16
Extended AAA-ATPase domain	1.57	2.2e-16
Subtilases	4.35	2.2e-16
Protein kinases, catalytic subunit	1.28	2.2e-16
G proteins	1.34	2.8e-16
Di-heme elbow motif	1.41	5.0e-16
V set domains (antibody variable-like)	1.31	4.8e-15
Glutathione S-transferase, C-terminal	1.60	6.7e-15
Most significant non-overlapping families		P-value (left)
Thi4-like	0.1	8.8e-12
Photosystem I subunits PsaA/PsaB	0.76	5.2e-10
Myeloperoxidase-like	0.23	6.5e-09
Heme-dependent catalases	0.47	1.0e-08
Eukaryotic type heme oxygenase	0.37	1.4e-08
Bacterial photosystem II (L and M)	0.84	2.0e-08
tRNA-guanine transglycosylase	0	1.3e-07
Catalase-peroxidase KatG	0.22	1.8e-07
Undecaprenyl diphosphate synthase	0.14	5.2e-07
MTH1675-like	0.09	7.9e-07

Bi-functional positions refer to alignment positions that bind both small molecules (250-1000 Da) and proteins. The significance of the overlap (Text Eqn 1) is assessed by the Fisher exact test (`http://r-project.org`).
