# Supplementary material for: The Overlap of Small Molecule and Protein Binding Sites within Families of Protein Structures
Source: PLoS Comput Biol. 2010 Feb 5;6(2):e1000668. doi: 10.1371/journal.pcbi.1000668 (PMC2816688; doi:10.1371/journal.pcbi.1000668)
Supplement: Table S3 — Summary of protein interactions and their overlap with aligned ligand binding sites from homologous structures. The numbers of protein interfaces with at least 20% cumulative or maximal overlap with homologous ligand binding sites are shown for each kind of protein interface. The overlap score refers to the fraction of interface residues aligned to ligand binding site residues (Text Eqn 2). (0.03 MB PDF) [file pcbi.1000668.s003.pdf]

Table S3: **Summary of protein interactions and their overlap with aligned ligand binding sites from homologous structures.**

		ligand overlap $\geq 20\%$	
Number of interactions	total	cumulative	maximal
Domain – peptide	2,332	1,215	1,070
Domain – domain (inter-chain)	12,015	5,617	4,170
Domain – domain (intra-chain)	4,290	2,053	1,498
Total	18,637	8,885	6,738

The numbers of protein interfaces with at least 20% cumulative or maximal overlap with homologous ligand binding sites are shown for each kind of protein interface. The overlap score refers to the fraction of interface residues aligned to ligand binding site residues (Text Eqn 2).
