# Supplementary material for: The Overlap of Small Molecule and Protein Binding Sites within Families of Protein Structures
Source: PLoS Comput Biol. 2010 Feb 5;6(2):e1000668. doi: 10.1371/journal.pcbi.1000668 (PMC2816688; doi:10.1371/journal.pcbi.1000668)
Supplement: Table S4 — The function propensities of families with significantly (p<0.01) higher or lower number of bi-functional positions than expected by chance. Bootstrap resampling was performed to compute 95% confidence intervals of the function propensities (Text Eqn 5). Propensities are considered significant (asterisk) at the alpha = 0.05 level if their confidence intervals do not include the value 1. (0.03 MB PDF) [file pcbi.1000668.s004.pdf]

Table S4: **The function propensities of families with significantly ($p < 0.01$) higher or lower number of bi-functional positions than expected by chance.**

Function	Propensity of families with significantly low bi-functional positions	(95% confidence interval)	Propensity of families with significantly high bi-functional positions	(95% confidence interval)
Information	1.197	(0.535, 2.123)	1.099	(0.571, 1.768)
Metabolism	1.237	* (1.074, 1.401)	0.848	* (0.719, 0.976)
Extracellular processes	0.325	(0, 1.051)	0.932	(0.193, 2.135)
Intracellular processes	0.718	(0.296, 1.252)	1.648	* (1.169, 2.298)
Regulation	0.39	* (0, 0.858)	1.118	(0.607, 1.765)
General	0.666	(0.204, 1.264)	1.464	(0.879, 2.205)
Other	0.337	* (0, 0.875)	0.387	* (0.084, 0.852)

Bootstrap resampling was performed to compute 95% confidence intervals of the function propensities (Text Eqn 5). Propensities are considered significant (asterisk) at the $\alpha = 0.05$ level if their confidence intervals do not include the value 1.
