# Supplementary material for: The Overlap of Small Molecule and Protein Binding Sites within Families of Protein Structures
Source: PLoS Comput Biol. 2010 Feb 5;6(2):e1000668. doi: 10.1371/journal.pcbi.1000668 (PMC2816688; doi:10.1371/journal.pcbi.1000668)
Supplement: Table S5 — The residue type propensity at alignment positions that bind both ligands and proteins, bind ligands, or bind proteins in comparison to all solvent-exposed residues. Bootstrap resampling was performed to compute 95% confidence intervals (CI) of the residue type propensities (Text Eqn 3). Propensities are considered significant (asterisk) at the alpha = 0.05 level if their confidence intervals do not include the value 1. (0.03 MB PDF) [file pcbi.1000668.s005.pdf]

Table S5: **The residue type propensity at alignment positions that bind both ligands and proteins, bind ligands, or bind proteins in comparison to all solvent-exposed residues.**

amino acid	Propensity at Ligand-only positions	(95% confidence) interval)	Propensity at Protein-only positions	(95% confidence interval)	Propensity at Bi-functional positions	(95% confidence interval)
A	1.027	(0.993, 1.06)	0.978	* (0.967 , 0.989)	0.943	* (0.919, 0.964)
C	1.425	* (1.334 , 1.529)	1.085	* (1.06 , 1.119)	1.002	(0.942, 1.053)
D	0.837	* (0.802 , 0.869)	0.996	(0.984 , 1.008)	0.932	* (0.909, 0.956)
E	0.685	* (0.658 , 0.712)	1.016	* (1.004 , 1.029)	0.831	* (0.811, 0.852)
F	1.367	* (1.312 , 1.424)	0.972	* (0.953 , 0.987)	1.178	* (1.139, 1.211)
G	1.277	* (1.236 , 1.316)	0.931	* (0.92 , 0.941)	1.095	* (1.072, 1.117)
H	1.332	* (1.267 , 1.394)	0.976	* (0.954 , 0.997)	1.179	* (1.139, 1.225)
I	1.172	* (1.131 , 1.217)	1.003	(0.99 , 1.018)	0.955	* (0.928, 0.98)
K	0.65	* (0.622 , 0.677)	1.002	(0.99 , 1.014)	0.904	* (0.882, 0.927)
L	1.103	* (1.074 , 1.136)	0.996	(0.985 , 1.007)	0.967	* (0.946, 0.988)
M	1.363	* (1.283 , 1.442)	1.012	(0.987 , 1.036)	1.114	* (1.071, 1.16)
N	0.912	* (0.872 , 0.952)	1.009	(0.993 , 1.024)	1.009	(0.983, 1.041)
P	0.748	* (0.711 , 0.783)	1.035	* (1.019 , 1.049)	0.893	* (0.867, 0.92)
Q	0.712	* (0.67 , 0.748)	1.024	* (1.009 , 1.041)	0.954	* (0.922, 0.981)
R	0.849	* (0.815 , 0.884)	1.04	* (1.027 , 1.055)	1.019	(0.992, 1.044)
S	0.925	* (0.892 , 0.962)	1.02	* (1.007 , 1.032)	1.059	* (1.032, 1.084)
T	0.971	(0.934 , 1.006)	1.007	(0.993, 1.021)	1.024	(0.998, 1.051)
V	1.152	* (1.113 , 1.187)	1.008	(0.997, 1.022)	0.978	(0.954, 1.003)
W	1.442	* (1.348 , 1.529)	0.946	* (0.92, 0.972)	1.33	* (1.267, 1.383)
Y	1.171	* (1.122 , 1.221)	0.991	(0.975, 1.01)	1.242	* (1.205, 1.28)

Bootstrap resampling was performed to compute 95% confidence intervals (CI) of the residue type propensities (Text Eqn 3). Propensities are considered significant (asterisk) at the $\alpha = 0.05$ level if their confidence intervals do not include the value 1.
