# Supplementary material for: The Overlap of Small Molecule and Protein Binding Sites within Families of Protein Structures
Source: PLoS Comput Biol. 2010 Feb 5;6(2):e1000668. doi: 10.1371/journal.pcbi.1000668 (PMC2816688; doi:10.1371/journal.pcbi.1000668)
Supplement: Table S6 — Examples of ligand binding sites that align to protein–protein interfaces with a high sequence similarity. The overlap (Text Eqn 2) between each ligand and protein interface is shown along with the sequence identity of the ligand binding site and the full-length domain sequence. (d) refers to inter-molecular domain–domain interactions, (p) refers to domain–peptide interactions, and (*) indicates ligands that were present at domain interfaces. (0.04 MB PDF) [file pcbi.1000668.s006.pdf]

Table S6: **Examples of ligand binding sites that align to protein–protein interfaces with a high sequence similarity.**

Protein interaction		Ligand	Overlap	Sequence identity bind site	Sequence identity domain
Enzyme–protein inhibitors					
1iid:A,O	(p) N-myristoyltransferase – GLYASKLA	1iyl:R64	63%	100%	52%
2q4g:Y,Z	(d) Ribonuclease I – human ribonuclease inhibitor	1u1b:PAX	42%	100%	64%
1bqq:M,T	(d) MT1-MMP–TIMP-2	1rm8:BAT	37%	100%	67%
1ta3:A,B	(d) Xylanase – inhibitor	1e0v:FFC	33%	100%	44%
1zli:A,B	(p) Carboxypeptidase B – tick inhibitor	1bav:BIP	21%	92%	45%
2f91:A,B	(p) Crayfish trypsin – peptide inhibitor	1y3y:UIR	48%	95%	41%
1y1k:E,I	(p) Subtilisin BPN – chymotrypsin inhibitor 2	1bh6:1BH	81%	93%	69%
2aq9:A,X	(p) UDP-N-Acetylglucosamine acyltransferase – peptide	1j2z:SOG	64%	86%	40%
Enzyme–protein substrates; Enzyme homodimers					
1tg4:A,I	(p) Russells Viper Phospholipase A2 – FLAYK	1rgb:ELD (*)	69%	100%	54%
1t4f:M,P	(p) MDM2 – p53 peptide	1ttv:IMY	100%	96%	62%
1qgd:A,B	(d) Transketolase dimer	1itz:TPP	37%	100%	42%
1fpy:D,E	(d) Glutamine synthetase dimer	2bvc:P3S	21%	100%	53%
1akm:A,C	(d) Ornithine transcarbamylase dimer	2fzc:EOP	20%	100%	34%
Regulatory or structural interaction					
2prg:A,C	(d) PPARγ – Src-1	1y0s:B7G	53%	100%	62%
1ib1:C,G	(d) 14-3-3 ζ – Seretonin N-Acetyltransferase	1o9e:FSC	40%	100%	63%
1a38:A,P	(p) 14-3-3 ζ – R18 peptide	1o9e:FSC	50%	100%	63%
1nwd:A,C	(p) Calmodulin – Glutamate decarboxylase (c-term)	1lxf:BEP (*)	52%	86%	34%
1k93:B,E	(d) Calmodulin – Anthrax edema factor	1lxf:BEP (*)	2%	86%	32%

The overlap (Text Eqn 2) between each ligand and protein interface is shown along with the sequence identity of the ligand binding site and the full-length domain sequence. (d) refers to inter-molecular domain–domain interactions, (p) refers to domain–peptide interactions, and (*) indicates ligands that were present at domain interfaces.
