# Supplementary material for: The Overlap of Small Molecule and Protein Binding Sites within Families of Protein Structures
Source: PLoS Comput Biol. 2010 Feb 5;6(2):e1000668. doi: 10.1371/journal.pcbi.1000668 (PMC2816688; doi:10.1371/journal.pcbi.1000668)
Supplement: Table S7 — Examples of ligand binding sites that align to intra-molecular domain–domain interfaces. The overlap (Text Eqn 2) between each ligand and domain interface is shown along with the sequence identity of the ligand binding site and the full-length domain sequence. (0.03 MB PDF) [file pcbi.1000668.s007.pdf]

Table S7: **Examples of ligand binding sites that align to intra-molecular domain–domain interfaces**

Interaction		Ligand	Overlap	Sequence identity bind site	Sequence identity domain
(A) Exact sequence matches					
2c78:A	Elongation factor Tu	1ha3:MAU	93%	100%	100%
1su3:B	ProMMP-1	4ayk:CGS	76%	100%	96%
4ake:A	Adenylate kinase	1ake:AP5	75%	100%	100%
1fmk:A	Tyrosine kinase c-Src	1o42:843	50%	100%	95%
1t56:A	TetR/CamR family repressor EthR	1u9n:CNS	38%	100%	99%
1xmm:D	DcpS	1st4:GTA	38%	95%	99%
1g6l:A	HIV Protease	1dif:A85	37%	100%	99%
1ft4:A	Tumor necrosis factor	1ft4:703	36%	100%	100%
1b3r:C	S-adenosylhomocysteine hydrolase	1li4:NOC	36%	94%	95%
2ofp:A	Ketopantoate reducase	1yon:APX	35%	100%	100%
1jt0:C	QacR–DNA complex	1jus:RHQ	32%	100%	100%
(B) Highly similar ligand binding sites					
1v8b:C	Adenosylhomocysteinase	1k0u:DEA	26%	93%	48%
1bg5:A	Alpha-Na K-ATPase – GST fusion	1c72:EPY	22%	93%	57%
2bvn:B	Elongation factor Tu	2c78:PUL	59%	93%	67%
1ak2:A	Adenylate kinase 2	1dvr:ATF	33%	93%	48%
2b8e:B	CopA ATP binding domain	1t5s:ACP	20%	93%	35%
1exm:A	Elongation Factor Tu	1ob2:KIR	79%	92%	68%
1gxd:B	72 KDa Type IV collagenase	830c:RS1	63%	90%	63%
1ad5:B	Haematopoetic cell kinase HCK	1fbz:CC1	56%	89%	68%
1miy:B	CCA-adding enzyme	1vfg:APC	26%	90%	30%

The overlap (Text Eqn 2) between each ligand and domain interface is shown along with the sequence identity of the ligand binding site and the full-length domain sequence.
