# Supplementary figures and images for: The Overlap of Small Molecule and Protein Binding Sites within Families of Protein Structures
Source: PLoS Comput Biol. 2010 Feb 5;6(2):e1000668. doi: 10.1371/journal.pcbi.1000668 (PMC2816688; doi:10.1371/journal.pcbi.1000668)

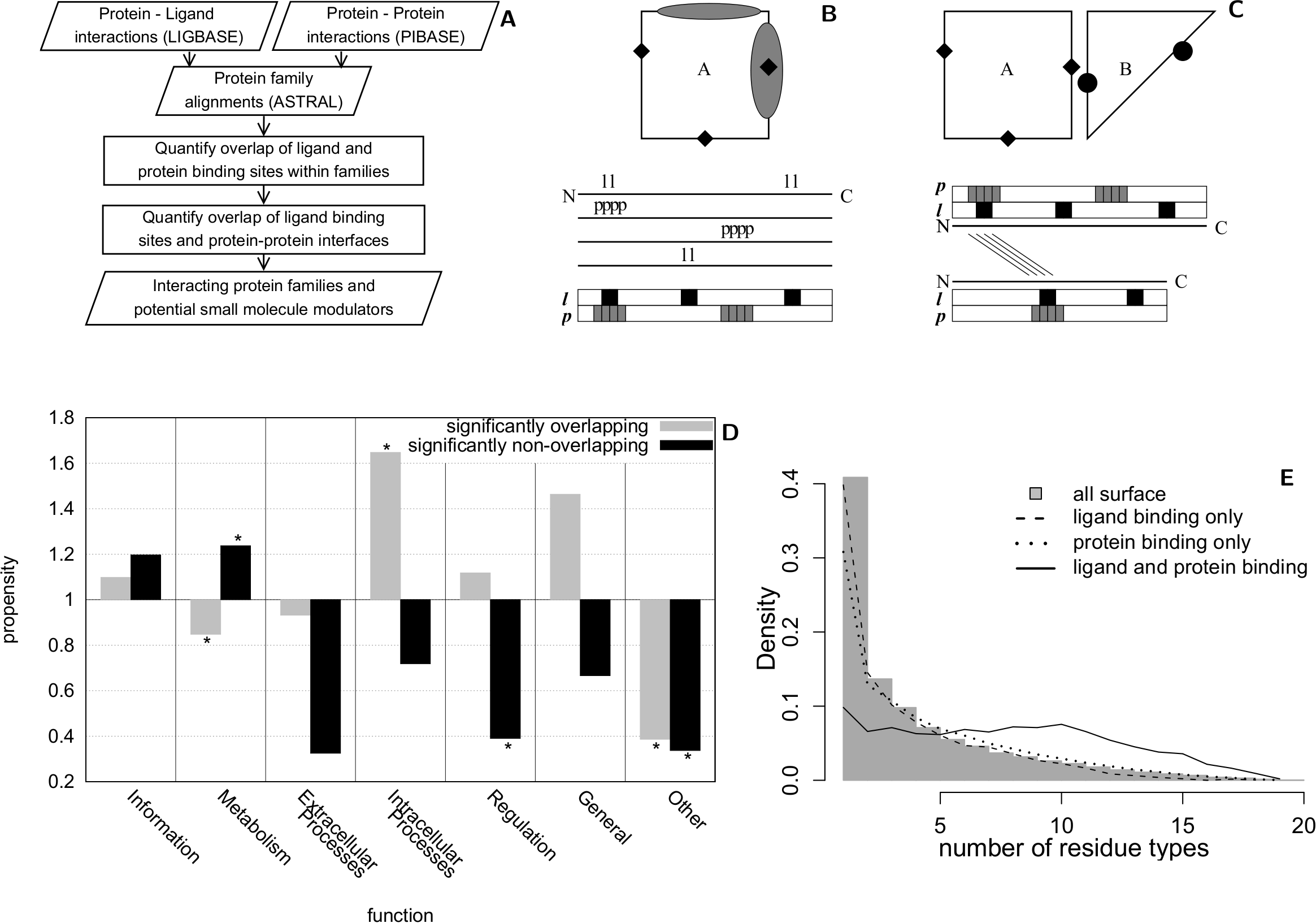

Supplement: Figure S1 — Protocol for quantifying binding site overlap, functional, and evolutionary properties. (A) Ligand and protein binding sites obtained from LIGBASE and PIBASE, respectively, were mapped onto domain family alignments from the SCOP ASTRAL compendium. (B) The square labeled A is a cartoon representation of a protein domain family upon which ligand (diamonds) and protein (grey ellipses) have been mapped. These binding sites are mapped onto the ASTRAL alignment of the family and the cumulative overlap of ligand and protein binding positions is quantified. (C) The ligand binding sites are also mapped directly onto individual protein interfaces, in this case the interaction between domains A and B, and the overlap quantified. (D) The distribution of function propensities (Text Eqn 5) for significantly overlapping and non-overlapping families, as annotated by SUPERFAMILY. Function propensities were considered significant (asterisk) at the alpha = 0.05 level if the 95% confidence interval estimated by bootstrap resampling did not include the value 1 (Table S4). (E) Residue conservation of bi-functional alignment positions. The number of amino acid types observed at alignment positions that are involved in binding only ligands (dashed; n = 46,610), only proteins (double dashed; n = 491,723), or both proteins and ligands (black;n = 102,436). The distribution for all solvent exposed residues (grey; n = 1,147,882) is shown for comparison. (0.34 MB TIF) [file pcbi.1000668.s008.tif]

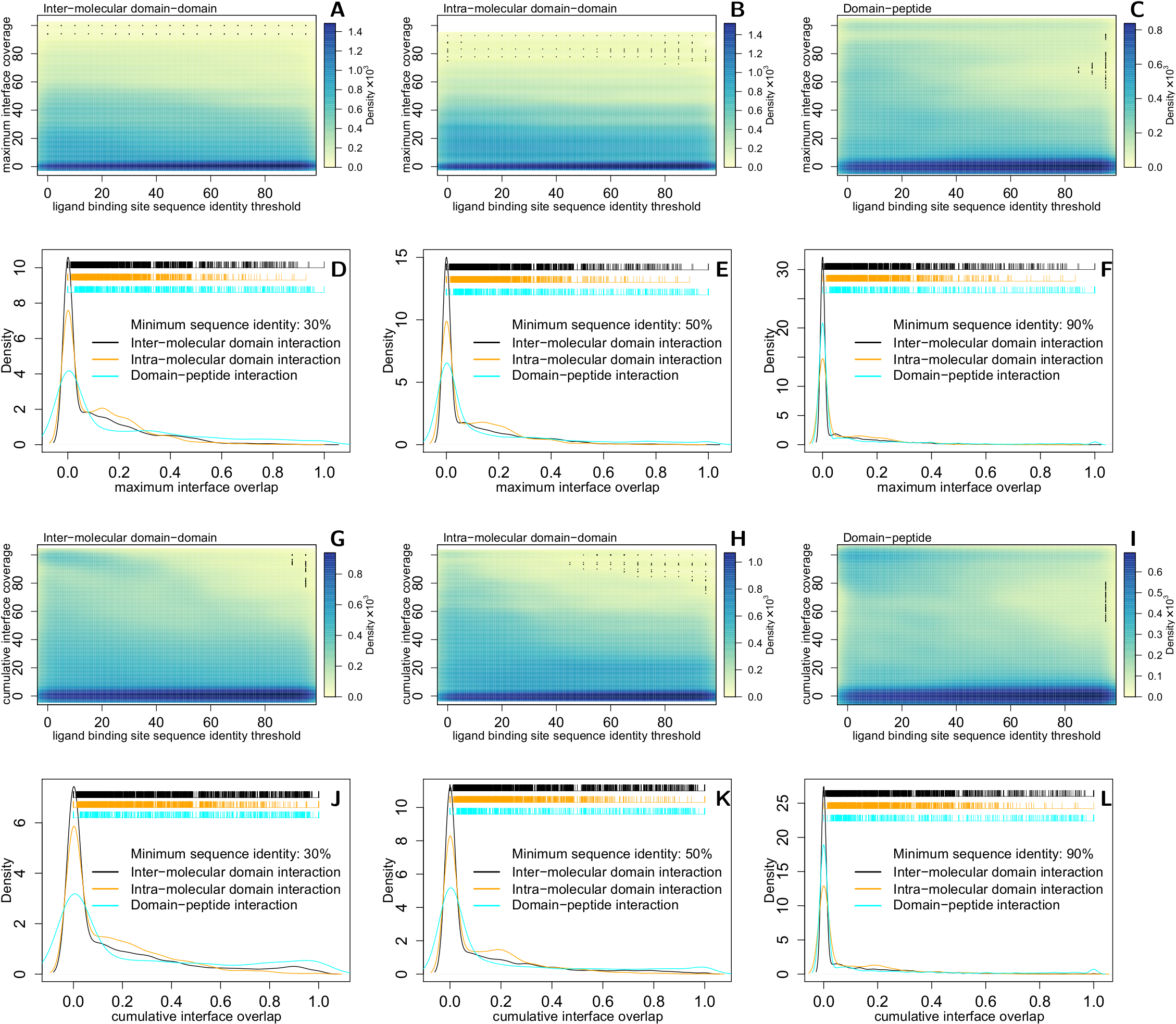

Supplement: Figure S2 — Maximum and cumulative ligand-protein binding site overlap observed at protein–protein interactions as a function of sequence identity. The maximum and cumulative observed ligand binding site overlap (y-axis) for (A,G) inter-molecular, (B,H) intra-molecular domain–domain, and (C,I) domain–peptide interactions, as a function of the ligand binding site sequence identity (x-axis). The densities in these plots are represented by colors that range from yellow (no density) to blue (maximum density). The overlap profiles are shown at minimum ligand binding site identity thresholds of (D,J) 30%, (E,K) 50%, and (F,L) 90% for inter-molecular (black), intra-molecular (orange) domain–domain, and domain–peptide (cyan) interactions. Tick marks, arranged as ‘rug plots’, represent interfaces of each type that exhibit a particular level of interface coverage. The overlap score refers to the fraction of interface residues aligned to ligand binding site residues (Text Eqn 2). (1.87 MB TIF) [file pcbi.1000668.s009.tif]
